# A facile way to obtain near-infrared room-temperature phosphorescent soft materials based on Bodipy dyes†
†Electronic supplementary information (ESI) available. See DOI: 10.1039/c9sc05502a


**DOI:** 10.1039/c9sc05502a

**Published:** 2019-11-25

**Authors:** Ting Zhang, Xiang Ma, He Tian

**Affiliations:** a Key Laboratory for Advanced Materials , Feringa Nobel Prize Scientist Joint Research Center , Institute of Fine Chemicals , School of Chemistry and Molecular Engineering , East China University of Science and Technology , Shanghai 200237 , China . Email: maxiang@ecust.edu.cn

## Abstract

Research on pure organic room-temperature phosphorescent (RTP) materials has made great advances but near-infrared (NIR) RTP emitting materials are still rare. Novel amorphous acrylamide copolymers containing iodine substituted borondipyrromethene (Bodipy) were prepared to obtain strong absorption in the visible region and moderate RTP in the NIR region with much larger Stokes shift than the fluorescence emission of traditional Bodipy dyes. Expensive metals and crystallization were left out to avoid biotoxicity and strict preparation conditions. Monoiodo and diiodo-Bodipy derivatives were both designed to study the substitution effect of iodine atoms. Photophysical properties, phosphorescence quantum yield and lifetime were characterized. Gels with NIR RTP emission were facilely prepared with the incorporation of ureidopyrimidone (UPy) and *N*,*N*′-methylenebisacrylamide (MBAA). The mechanical properties of the gels were measured using a rheometer and the results showed that the gels displayed fast self-healing ability due to the strong quadruple hydrogen bonding between UPy moieties.

## Abstract 2

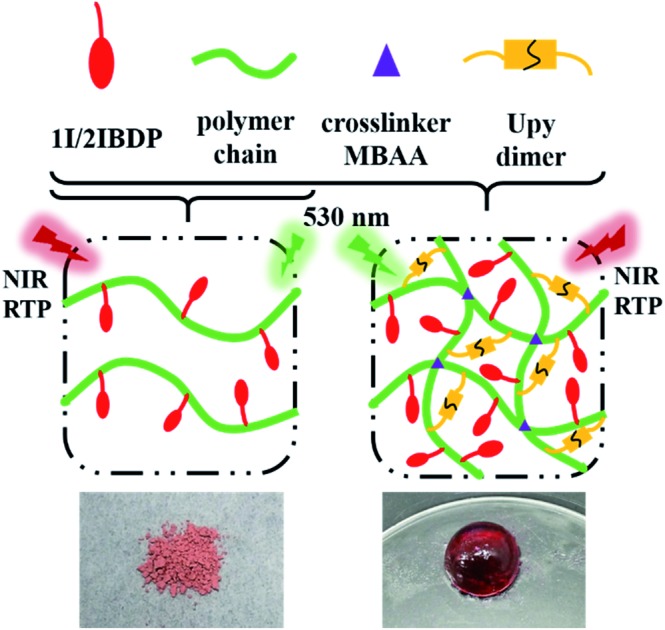
Near-infrared room-temperature phosphorescence was achieved by employing iodine substituted Bodipy into amorphous polymers. The self-healable gels were also obtained with the incorporation of a crosslinker and quadruple hydrogen bond based moieties.

## Introduction

Room-temperature phosphorescence (RTP) emitting materials have been one of the most extensively investigated luminescent materials in recent years due to their distinctive photophysical properties and wide applications in various fields including bioimaging,^1^–^4^ organic light-emitting diodes,^5^,^6^ molecular switches,^7^,^8^ and so forth. Compared with traditional metal complexes and inorganic compounds, pure organic materials with RTP emission are gradually arousing people's interest because of their low cost and low toxicity.^9^–^12^ In particular, great achievements have been made in preparing RTP emitting materials in the amorphous state because crystallization usually needs strict growth conditions which might restrict their development.^13^–^18^ Amorphous polymers have been verified to be rigid enough to effectively suppress nonradiative transition and promote RTP emission.^19^–^26^ Various phosphors with different conjugated structures have been utilized to obtain RTP emission of different colours, most of which are excited by UV light and exhibit RTP emission in the visible region.^27^–^31^ Materials with absorption in the visible region and emission in the NIR region are promising candidates in a wide range of fields, such as medical and therapeutic applications, due to their high tissue penetration and low autofluorescence.^32^–^35^ Up till now, materials with NIR RTP emission have been essentially limited to metal complexes like Pt and Ir, which suffer from complicated synthesis, high cost and biotoxicity.^36^–^38^ Therefore, it's desirable and worthwhile to develop pure organic phosphors with NIR phosphorescence emission. Boron dipyrromethene (Bodipy) has long been a widely used chromophore with a large molar absorption coefficient, high fluorescence quantum yield and good photostability.^39^–^42^ Since traditional Bodipy dyes generally suffer from small Stokes shift which restricts their applications in biomedical areas because of the disturbance of background fluorescence, it would be promising to study the phosphorescence emission of Bodipy dyes with larger Stokes shift. In the last few years, the heavy atom effect has been utilized to promote the intersystem crossing (ISC) efficiency of Bodipy by enhancing spin–orbital coupling and NIR RTP has been achieved at low temperature.^43^–^45^ The efficient triplet generation of Bodipy dyes is gradually attracting people's attention due to their potential applications in photodynamic therapy, light harvesting, chemosensors and triplet–triplet annihilation (TTA) based upconversion.^46^–^49^ However, reports of phosphorescence emission of metal-free Bodipy derivatives are still rare and are restricted to low temperature or deoxidized conditions.^50^–^53^

**Scheme 1:**
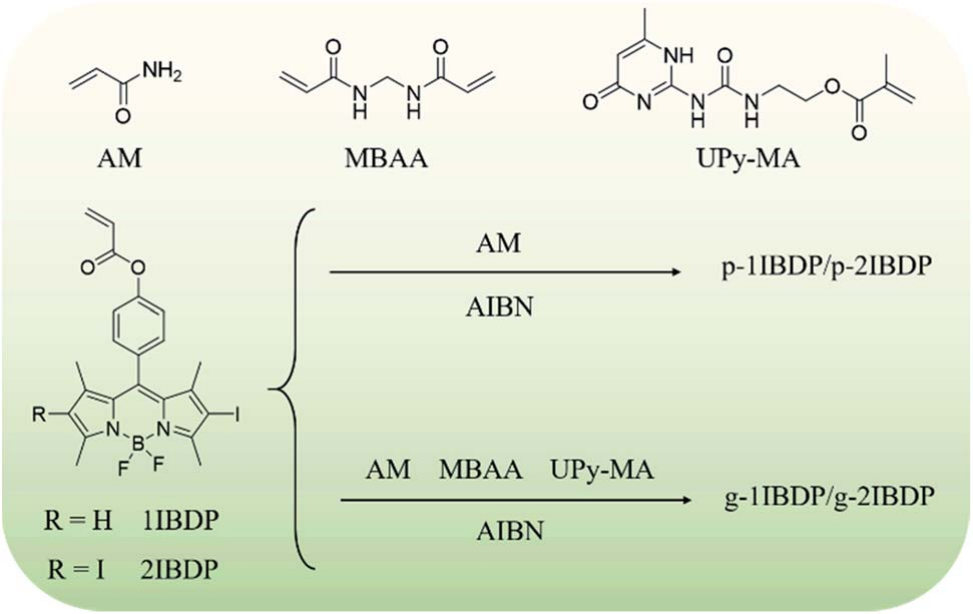
Synthetic route of the polymers.

Herein, two pure organic NIR RTP emitting materials in the amorphous state are facilely prepared by radical binary copolymerization of acrylamide and iodine-substituted Bodipy dyes (Scheme 1). The heavy atom effect of iodine can enhance the spin–orbital coupling and facilitate the ISC process. Monoiodo and diiodo-Bodipy derivatives have both been synthesized to investigate the substitution effect of iodine atoms. Copolymerizing acrylamide and different phosphors has been proved to be an efficient way to immobilize the phosphors and achieve RTP of different emission colours.^18^,^24^,^25^ The rigid matrix of a polyacrylamide system can protect phosphors from the quenching effect of oxygen molecules and restrict their nonradiative transition. The two polymers (p-1IBDP and p-2IBDP) in this research work display strong absorption in the visible region and moderate RTP in the NIR region with a large Stokes shift of up to 250 nm. To the best of our knowledge, this is the first report of pure organic amorphous materials exhibiting RTP emission in the NIR region with no need for deaeration. Moreover, by incorporating a crosslinker *N*,*N*′-methylenebisacrylamide (MBAA) and ureidopyrimidone (UPy) moieties into the polymerization process, self-healable NIR RTP emitting gels were obtained. UPy moieties can form stable dimers through quadruple hydrogen bonding which is extremely strong with an outstanding association constant (around 6 × 10^7^ M^–1^ in CHCl_3_).^54^ The strong quadruple hydrogen bonding endows the gels with fast self-healing ability.

## Results and discussion

Copolymerizing acrylamide and pure organic phosphors has been demonstrated to be an efficient and simple way to engender RTP emission. The rigid matrix of polyacrylamide can effectively immobilize the phosphors and inhibit the nonradiative transition process. Different colours of RTP including blue-purple, green, and orange have been achieved by employing several different phosphors.^18^,^24^,^25^ In order to extend the RTP emission colour to red and even the NIR region, polymers composed of acrylamide and iodine-substituted Bodipy dyes are designed in this research work. Scheme S1–S3† present the detailed synthetic route of the phosphorescent polymers. Alkenyl groups were first modified on Bodipy for further polymerization reaction with acrylamide. Then iodine atoms with lone pair electrons were introduced on the Bodipy core to promote the ISC process from the singlet to triplet excited state by spin–orbital coupling. To further investigate the influence of the number of iodine substituents, 2-iodo-Bodipy and 2,6-diiodo-Bodipy were designed and synthesized. The polymers were finally prepared by radical binary copolymerization of acrylamide and the two Bodipy derived monomers respectively with 2,2′-azobis(2-methylpropionitrile) (AIBN) as the radical initiator. With the incorporation of UPy and MBAA in the polymerization reaction, fast self-healable gels with NIR RTP emission were obtained. MBAA functions as a cross linker and the strong quadruple hydrogen bonding between UPy moieties endows the gels with fast self-healing capacity.

Spectral characteristics of the polymer monomers (1IBDP and 2IBDP) were studied using UV-Vis absorption spectra and fluorescence spectra. UV-Vis absorption spectra in diluted dichloromethane show that both 1IBDP and 2IBDP have strong sharp absorption peaks over 500 nm. Upon excitation at their maximum absorption wavelengths (518 nm and 535 nm), 1IBDP and 2IBDP showed fluorescence emission peaks at 535 nm and 555 nm, respectively (Fig. S1†). Similar to traditional Bodipy dyes, their fluorescence Stokes shifts are only around 20 nm. 2IBDP exhibits absorption and emission bands at longer wavelength than 1IBDP because of the different number of halogen substitution. Phosphorescence signal was not detected in the solution of 1IBDP and 2IBDP at room temperature because the triplet excited can be easily quenched by oxygen molecules and the nonradiative vibration and collision relaxation process.

**Fig. 1:**
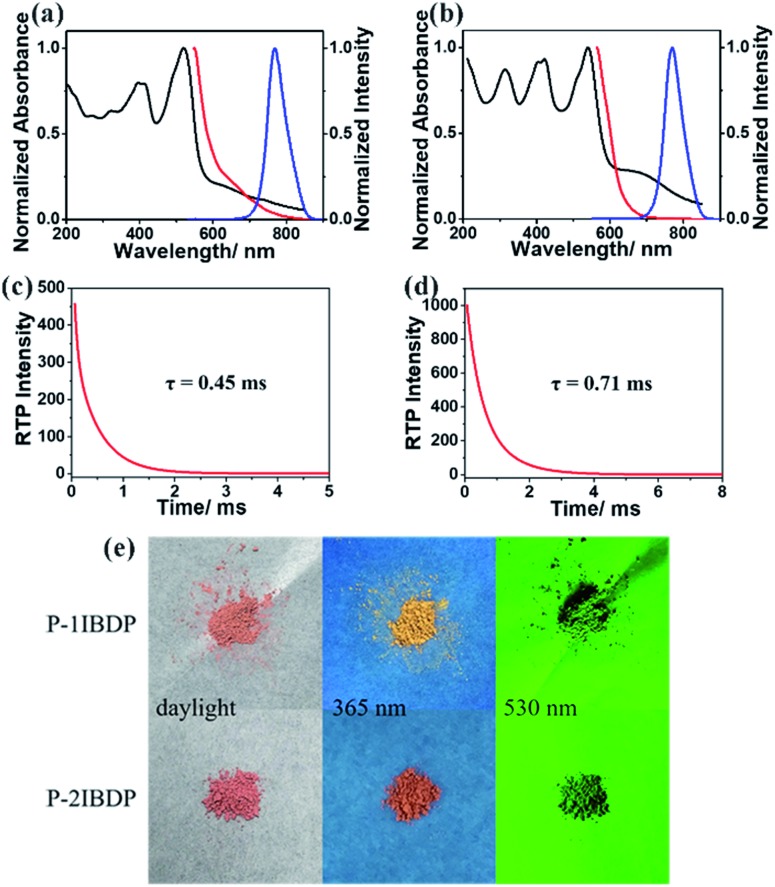
Photophysical properties of p-1IBDP and p-2IBDP. Normalized absorption (black), fluorescence (red) and RTP spectra (blue) of (a) p-1IBDP and (b) p-2IBDP in the amorphous solid state. Phosphorescence decay lifetimes of (c) p-1IBDP and (d) p-2IBDP at 770 nm in the amorphous solid state (*λ*_ex1_ = 520 nm, *λ*_ex2_ = 540 nm). (e) Photographs of p-1IBDP and p-2IBDP under different lights.

After copolymerization with acrylamide, amorphous powders p-1IBDP and p-2IBDP were obtained. UV-Vis absorption spectra of the polymers in the solid state are slightly different from the polymer monomers in diluted solutions (Fig. 1a and b). P-1IBDP shows two typical absorption peaks at around 520 nm and 400 nm as well a relatively weak one at 320 nm, while p-2IBDP exhibits three typical absorption peaks at 540 nm, 420 nm and 315 nm. Upon excitation at 520 nm, p-1IBDP engendered a fluorescence emission peak at 545 nm in the prompt photoluminescence (PL) spectra and a phosphorescence emission peak at 770 nm in the delayed PL spectra (delay time = 0.1 ms, gate time = 2.0 ms), demonstrating a small Stokes shift in the prompt PL spectra and a large Stokes shift in the delayed PL spectra. Similar results were obtained when p-2IBDP was excited with 540 nm. Fluorescence at 565 nm and phosphorescence at 770 nm were detected in prompt and delayed PL spectra, respectively. The delayed PL spectra of the two polymers demonstrate emission peaks at 770 nm, which were out of the range of visible light and appeared black under 530 nm light irradiation by the naked eye. Furthermore, lifetimes of p-1IBDP and p-2IBDP at 770 nm were characterized and calculated to be 0.45 ms and 0.71 ms (Fig. 1c and d). Phosphorescence has much longer lifetime than fluorescence due to the spin-forbidden process of ISC. Additionally, phosphorescence shows a longer emission wavelength compared with fluorescence because the energy level of the first triplet excited state is lower than that of the first singlet excited state. Both the large Stokes shift and long lifetime proved that the emission peak at 770 nm should be attributed to phosphorescence emission from the triplet excited state. The fluorescence Stokes shifts of the two polymers were less than 30 nm, while phosphorescence Stokes shifts reached up to 250 nm, which was rarely reported. Photographs of p-1IBDP and p-2IBDP under daylight, 365 nm UV light and 530 nm light were taken (Fig. 1e). Both of the polymers exhibit their fluorescence emission under 365 nm light and phosphorescence emission under 530 nm.

The heavy atom effect generally plays a key role in promoting ISC by enhancing spin–orbital coupling and inducing a triplet excited state. In this research work, p-1IBDP, p-2IBDP and a reference polymer p-BDP with no iodine substitution (Scheme S2†) were designed to investigate the heavy atom effect with different numbers of iodine substitutions. UV-Vis absorption spectra of p-BDP show the maximum absorption peak at 500 nm (Fig. S2†). Compared to p-1IBDP and p-2IBDP, p-BDP exhibits extremely strong fluorescence emission and negligible phosphorescence emission due to the intrinsic high fluorescence quantum yield of Bodipy dyes and lack of heavy atom effect.

**Fig. 2:**
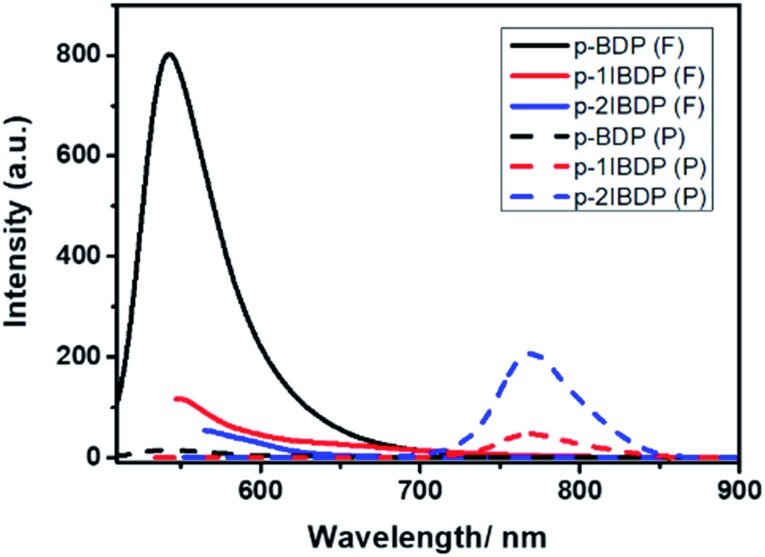
Fluorescence and RTP emission spectra of p-BDP, p-1IBDP and p-2IBDP (*λ*_ex_ = 500 nm, *λ*_ex1_ = 520 nm, *λ*_ex2_ = 540 nm).

With more iodine substituents on the Bodipy core, p-2IBDP exhibits weaker fluorescence and stronger phosphorescence (Fig. 2). It is clear that the iodination of the Bodipy core shows a remarkable effect on fluorescence and phosphorescence intensity because of the enhanced S_1_–T_1_ ISC caused by the heavy atom effect. All the photophysical properties, including phosphorescence quantum yields, are listed in Table 1.

**Table 1:** Photophysical properties of p-BDP, p-1IBDP, p-2IBDP, g-1IBDP and g-2IBDP. (f: fluorescence, p: phosphorescence.)

Compound	*λ*_ex_ [nm]	*λ*_em(f)_ [nm]	*λ*_em(p)_ [nm]	*τ*_p_ [ms]	*Φ* [%]
p-BDP	500	542	—	—	
p-1IBDP	520	545	770	0.45	0.4
p-2IBDP	540	565	770	0.71	0.5
g-1IBDP	520	550	775	0.063	0.1
g-2IBDP	540	570	775	0.083	0.2

**Fig. 3:**
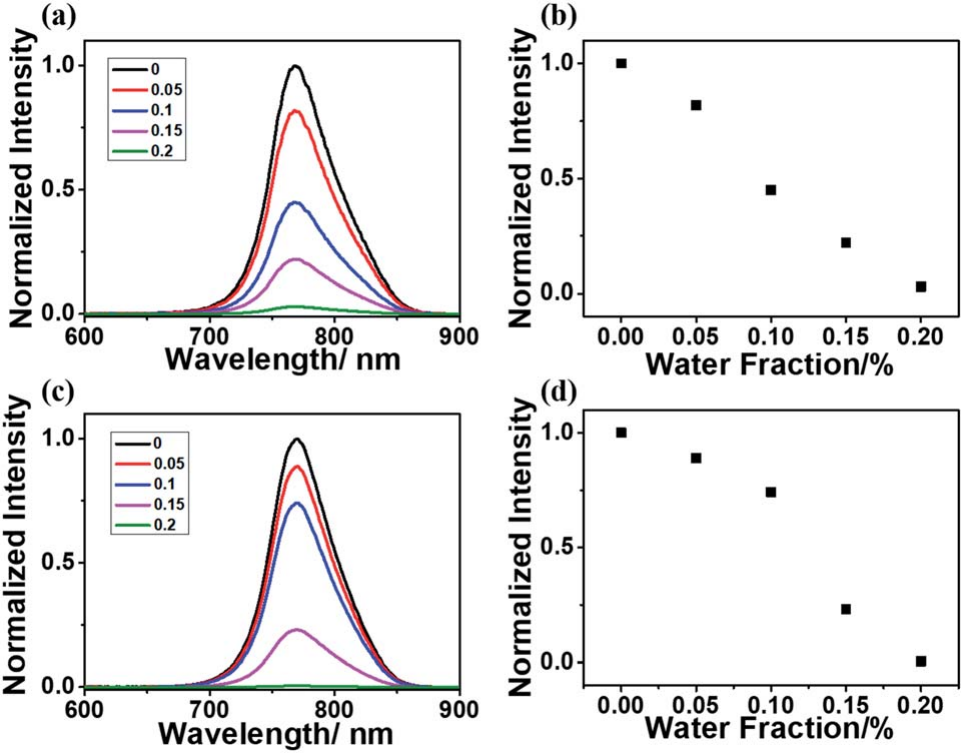
Normalized RTP spectra of (a) p-1IBDP and (c) p-2IBDP with various water fractions in a water/DMF solution mixture (*c* = 2 mg mL^–1^). Normalized RTP of (b) p-1IBDP and (d) p-2IBDP at 770 nm in the water/DMF solution mixture. (*λ*_ex1_ = 520 nm, *λ*_ex2_ = 540 nm).

To further verify the importance of the rigid matrix in achieving RTP emission, a DMF/H_2_O mixed solvent was utilized since the acrylamide polymers were only soluble in water and insoluble in most organic solvents. With the existence of water, the rigid matrix of polyacrylamide and the hydrogen bonding inside the polymeric chains will be broken, which will enhance the molecular vibration and lead to oxygen quenching effects. Both P-1IBDP and p-2IBDP were suspended in the mixed solvent with different water fractions. With the increase of water fraction in the mixed solvent, RTP of the two polymers decreased remarkably and was totally quenched when water fraction reached 0.2% (Fig. 3).

The humidity response of p-1IBDP and p-2IBDP makes them potential candidates in water content detection. X-ray powder diffraction (XRD) analysis was performed to verify the amorphous state of p-1IBDP and p-2IBDP. Similar to the pure polyacrylamide,^28^ the diffraction patterns of the two polymers showed no significant crystal characteristics, proving their amorphous states (Fig. S3†).

**Fig. 4:**
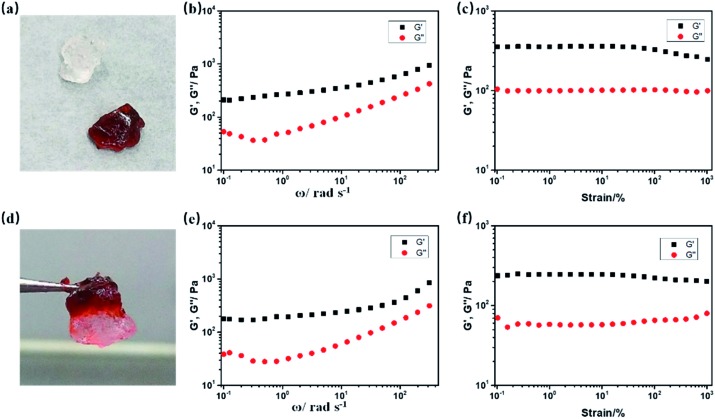
Mechanical properties and self-healing behaviour of the gels. (a) Photograph of the separate g-2IBDP and transparent gel. (d) Photograph of the self-healable gels. *G*′ and *G*′′ of (b) g-2IBDP and (e) the integral gel after the self-healing process as a function of scanning frequency (*ω*) at 25 °C and 0.5% strain amplitude. Strain sweep measurements of (c) g-2IBDP and (f) integral gel after the self-healing process at 25 °C (*G*′ and *G*′′ as a function of strain *γ*, *ω* = 10 rad s^–1^).

Since artificial soft materials like supramolecular gels are receiving wide attention,^55^,^56^ self-healable NIR RTP emitting gels were facilely prepared with the incorporation of UPy and MBAA into p-1IBDP and p-2IBDP. MBAA acts as a crosslinker. UPy moieties can form self-complementary dimers *via* quadruple hydrogen bonding interaction and have been utilized to prepare self-healable polymer gels.^57^–^61^ The feeding ratio of 1IBDP/2IBDP, acrylamide, UPy-MA and MBAA is 1 : 100 : 2.5 : 2. The two gels containing 1IBDP and 2IBDP are simplified as g-1IBDP and g-2IBDP, respectively. The obtained gels exhibit fast self-healing ability as shown in Fig. 4. A piece of g-2IBDP and a piece of transparent gel with no addition of Bodipy were put together for better discrimination. After putting together for only one minute, the two gels adhered to each other to form an integral gel and an intermediate colour was clearly shown on the interface between them (Fig. 4a and d). Rheological tests were performed on g-2IBDP and the healed gel after the self-healing process to investigate their mechanical properties. As shown in the strain sweep measurement, the values of storage modulus (*G*′) are distinctly larger than loss modulus (*G*′′), verifying the formation of the gels. Both g-2IBDP and the healed gel show constant *G*′ and *G*′′ from 0.1% to 100%. With the applied strain increasing up to 1000%, no crossover point is observed between *G*′ and *G*′′, suggesting that the gels could withstand large deformations from damage (Fig. 4c and f). The scanning frequency (*ω*) dependence of *G*′ and *G*′′ also exhibits typical elastic gel behaviour of the samples (Fig. 4b and e). The rheological data of the gels before and after the self-healing process show no obvious difference, which can confirm their excellent self-healing properties. In order to verify that the self-healing ability is ascribed to UPy moieties, a reference gel composed of 2IBDP, acrylamide and MBAA (1 : 100 : 2) was prepared. Without the strong hydrogen bonding between UPy, two pieces of the reference gels could still be easily separated using tweezers after being put together for 24 hours, showing no self-healing ability (Fig. S4†).

**Fig. 5:**
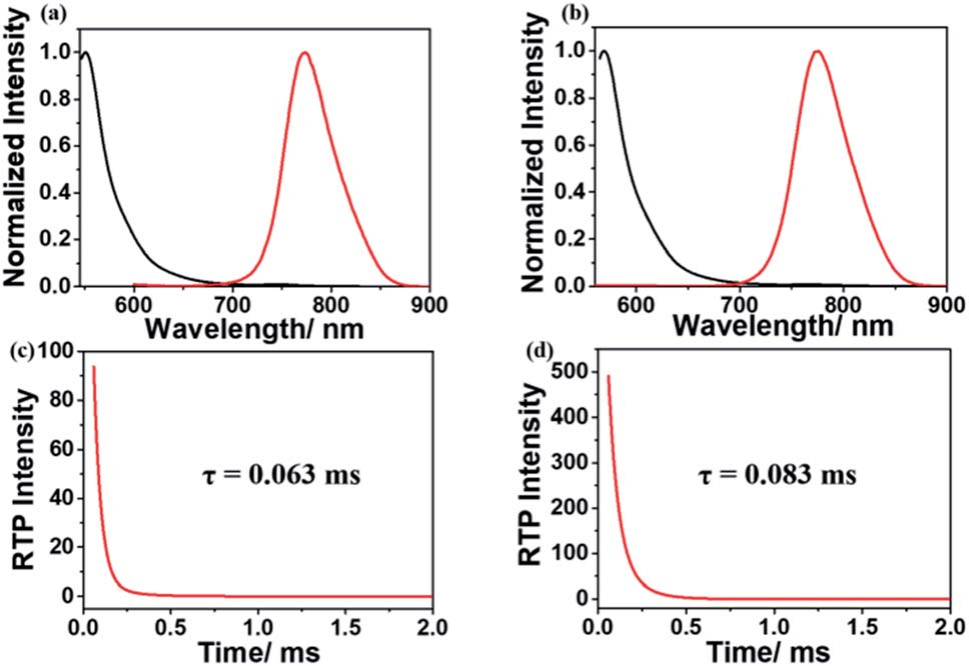
Photophysical properties of g-1IBDP and g-2IBDP. Normalized fluorescence (black) and RTP (red) of (a) g-1IBDP and (b) g-2IBDP. Phosphorescence decay lifetimes of (c) g-1IBDP and (d) g-2IBDP at 775 nm (*λ*_ex1_ = 520 nm, *λ*_ex2_ = 540 nm).

As shown in Fig. 5, fluorescence and RTP emission of g-1IBDP and g-2IBDP were all observed. The phosphors are well immobilized during the polymerization process to reduce nonradiative transition. Both the covalent bond and quadruple hydrogen bond inside the polymer gels have the rigidity effect and help to protect the phosphors from the quenching effect of oxygen molecules. The photoluminescence spectra of gels are similar to those of polymer powders. Upon excitation at 520 nm and 540 nm, fluorescence emissions of g-1IBDP and g-2IBDP appeared at 550 nm and 570 nm, while RTP emissions of both appeared at 775 nm. The two gels are red under daylight and seem black under 530 nm light by the naked eye because the emission band at 775 nm is out of the range of visible light. Furthermore, RTP lifetimes of g-1IBDP and g-2IBDP at 775 nm were characterized and calculated to be 0.063 ms and 0.083 ms (Fig. 5c and d).

The RTP lifetimes of the gels were shorter than those of the polymer powders because the solvent molecules were embedded inside the gels, which might weaken the polymer rigidity to some extent and enhance nonradiative transition. However, the lifetimes of the gels were still long enough to verify their triplet excited states. Phosphorescence excitation spectra of g-1IBDP and g-2IBDP were also measured to confirm the validity of RTP emission spectra (Fig. S5†). When comparing the relative intensity of fluorescence and RTP, the polymer gels showed the same results as the polymer powders. More iodine substituents on the Bodipy core lead to weaker fluorescence and stronger phosphorescence (Fig. S6†), demonstrating that the ISC process is promoted with a stronger heavy atom effect. All the photophysical properties of g-1IBDP and g-2IBDP, along with their phosphorescence quantum yields, are listed in Table 1.

## Conclusions

In summary, two pure organic amorphous polymers (p-1IBDP and p-2IBDP) composed of acrylamide and iodine substituted Bodipy dyes were designed. The preparation of the polymers is facile with high yields. Both of the polymers have strong absorption in the visible region and RTP emissions at 770 nm in the amorphous state, which is the first report of pure organic RTP emission in the NIR region. The phosphors are immobilized and isolated from oxygen molecules by the rigid polymer matrix to reduce nonradiative transition. The number of iodine substitutions on the Bodipy core remarkably influences the fluorescence and RTP emission intensity. It has been proved that more iodine substitution leads to more efficient ISC, along with weaker fluorescence and stronger phosphorescence. Polymer gels with NIR RTP emission were also successfully obtained with the addition of MBAA and UPy moieties. The gels exhibit fast self-healing capacity because of the strong quadruple hydrogen bonding between UPy moieties. This study would be beneficial for the future design of multifunctional and smart RTP materials with potential applications in various fields.

## Conflicts of interest

The authors declare no competing financial interests.

## Supplementary Material

Supplementary information
