# Supplementary material for: A facile way to obtain near-infrared room-temperature phosphorescent soft materials based on Bodipy dyes
Source: Chem Sci. 2019 Nov 25;11(2):482–7. doi: 10.1039/c9sc05502a (PMC7067252; doi:10.1039/c9sc05502a)
Supplement: Supplementary file 1 [file SC-011-C9SC05502A-s001.pdf]

Supporting Information

A Facile Way to Obtain Near-Infrared Room-Temperature Phosphorescent Soft Materials based on Bodipy Dyes

Ting Zhang, Xiang Ma* and He Tian

Key Laboratory for Advanced Materials and Feringa Nobel Prize Scientist Joint Research Center, Institute of Fine Chemicals, School of Chemistry and Molecular Engineering, East China University of Science and Technology, Shanghai 200237, China

Corresponding Author

*maxiang@ecust.edu.cn

Experimental Procedures

Materials: All reagents used in this research work were commercially available. AIBN (2,2'-azobis(2-methylpropionitrile)) was used after recrystallization in ethanol and the other reagents were all used without further purification. Solvents were purified according to standard laboratory methods. The in-termediates were confirmed by ^{1}H NMR, ^{13}C NMR and high resolution ESI mass spectroscopy. The solid polymers were characterized by aqueous gel permeation chromatography.

Characterization: ^{1}H NMR and ^{13}C NMR spectra were recorded on a Brüker AV-400 spectrometer with chemical shifts reported as ppm. The electronic spray ionization (ESI) high-resolution mass spectra were tested on a Waters LCT Premier XE spectrometer. UV-Vis spectra were done on a UV-2550 UV-Vis spectrophotometer and a Varian Lambda 950. Photoluminescence spectra and phosphorescence lifetimes were obtained on a Varian Cary Eclipse spectrophotometer and a Horiba Fluoremax-4 spectrofluorometer. Phosphorescence and delayed fluorescence were recorded in a delayed mode (delay time = 0.1 ms, gate time = 2.0 ms) on the Varian Cary Eclipse spectrophotometer. Quantum yields were measured by using an integrating sphere on a HAMAMATSU Quantaurus-QY C11347-11. Powder X-ray diffraction (XRD) was performed on a D/max2550V. Aqueous gel permeation chromatography (GPC) was measured on a Waters 2695. Rheological properties were measured using a TA Discovery HR-3 rheometer. Photographs were taken with a digital camera.

Synthetic routes

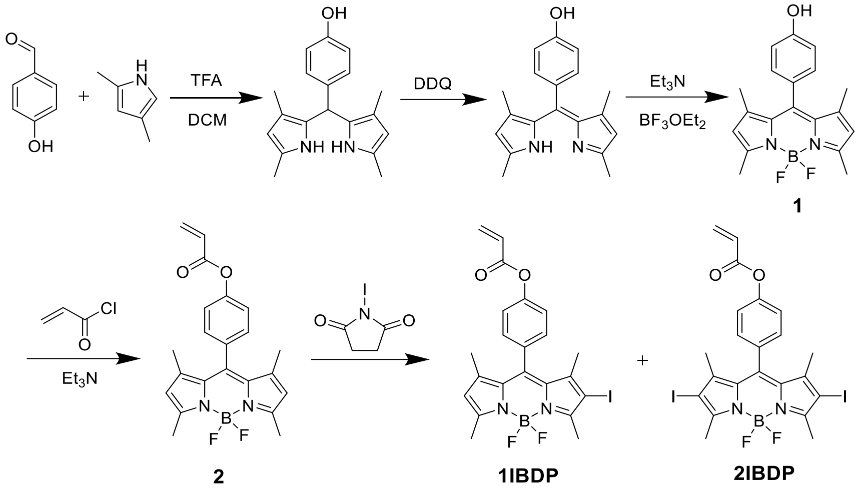

Scheme S1. Preparation routine of 1IBDP and 2IBDP.

Synthesis of Compound 1. Compound 1 was synthesized according to literature procedure with same structure.[S1]

Synthesis of Compound 2. Compound 1 (250 mg, 0.74 mmol, 1 eq) and trimethylamine (0.15 mL, 1.11 mmol, 1.5 eq) were firstly dissolved in dried dichloromethane under argon atmosphere and cooled with ice-water bath. Acryloyl chloride (0.071 mL, 0.89 mmol, 1.2 eq) was then added dropwise with a syringe. The mixture was stirred at 0 ^{o}C for 30 mins and room temperature for 9 hours. After reaction, the solution was washed with H_2O (3×15 mL) and brine (3×15 mL). The obtained organic phase was dried with anhydrous Na_2SO_4 and concentrated to give a red solid, which was purified by column chromatography (petroleum ether/ dichloromethane, 1/2) (236 mg, 82% yield). ^{1}H NMR (400 MHz, $CDCl_3$) δ 7.31 (d, J = 2.8 Hz, 4H), 6.65 (d, J = 16.2 Hz, 1H), 6.35 (dd, J = 16.2 Hz, 10.4 Hz, 1H), 6.07 (d, J = 10.4 Hz, 1H), 5.99 (s, 2H), 2.56 (s, 6H), 1.44 (s, 6H). ^{13}C NMR (100 MHz, $CDCl_3$) δ 163.11, 154.65, 150.10, 142.07, 139.59, 132.01, 131.44, 130.40, 128.13, 126.63, 121.43, 120.31, 28.66, 13.50. HR-ESI-MS m/z: $[M+H]^+$ calculated for $C_{22}H_{22}BF_2N_2O_2$ 395.1742; found, 395.1738.

Synthesis of 1IBDP and 2IBDP. Compound 2 (200 mg, 0.51 mmol, 1 eq) and N-iodosuccinimide (288 mg, 1.28 mmol, 2.5 eq) were dissolved in dried dichloromethane. After stirring at room temperature for 5 hours, the solution was washed with H_2O (3×15 mL) and brine (2×15 mL). The organic phase was dried

with anhydrous Na_2SO_4 and concentrated to give a mixture of 1IBDP and 2IBDP. Column chromatography were used to separate them (petroleum ether/ dichloromethane, 2/1). 1IBDP (145 mg, 55% yield). ^{1}H NMR (400 MHz, $CDCl_3$) δ 7.31 (d, J = 1.7 Hz, 4H), 6.66 (d, J = 17.3 Hz, 1H), 6.36 (dd, J = 17.3, 10.4 Hz, 1H), 6.07 (d, J = 10.4 Hz, 1H), 6.06 (s, 1H) 2.64 (s, 3H), 2.57 (s, 3H), 1.45 (s, 6H). ^{13}C NMR (100 MHz, $CDCl_3$) δ 164.11, 158.06, 154.81, 151.35, 145.13, 140.43, 133.18, 132.31, 129.13, 127.65, 122.68, 122.48, 16.86, 15.83, 14.81. HR-ESI-MS m/z: $[M+K]^+$ calculated for $C_{22}H_{20}BF_2IN_2O_2K$ 559.0268; found, 559.0278. 2IBDP (115 mg, 35% yield). ^{1}H NMR (400 MHz, $CDCl_3$) δ 7.36-7.27 (m, 4H), 6.66 (d, J = 17.3, 1H), 6.36 (dd, J = 17.3 Hz, 10.4 Hz, 1H), 6.08 (d, J = 10.4, 1H), 2.65 (s, 6H), 1.45 (s, 6H). ^{13}C NMR (100 MHz, $CDCl_3$) δ 164.04, 157.05, 151.55, 145.34, 140.30, 133.27, 132.18, 131.35, 129.06, 127.60, 122.87, 85.89, 29.73, 17.19, 16.08. HR-ESI-MS m/z: $[M-H]^-$ calculated for $C_{22}H_{18}BF_2I_2N_2O_2$ 644.9519; found, 644.9523.

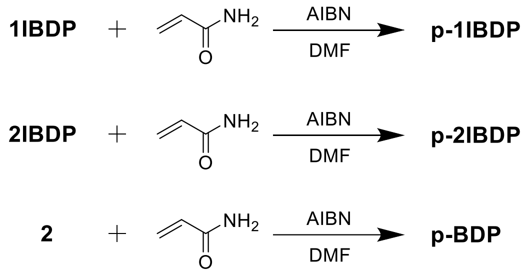

Scheme S2. Preparation routine of p-1IBDP, p-2IBDP and p-BDP.

Synthesis of p-1IBDP, p-2IBDP and p-BDP. These polymers were prepared by copolymerization of 1IBDP/2IBDP/**2** (1 eq) and acrylamide (100 eq) by radical polymerization with AIBN (0.1 eq) as the radical initiator at 70 ^oC under argon atmosphere in 1.5 mL DMF for 15 h. The precipitated solid was repeatedly washed with methanol to give p-1IBDP, p-2IBDP and p-BDP. GPC (H_2O, p-1IBDP): Mn (PDI) = 9547 Da (3.49); GPC (H_2O, p-2IBDP): Mn (PDI) = 5987 Da (2.52); GPC (H_2O, p-IBDP): Mn (PDI) = 7441 Da (3.05).

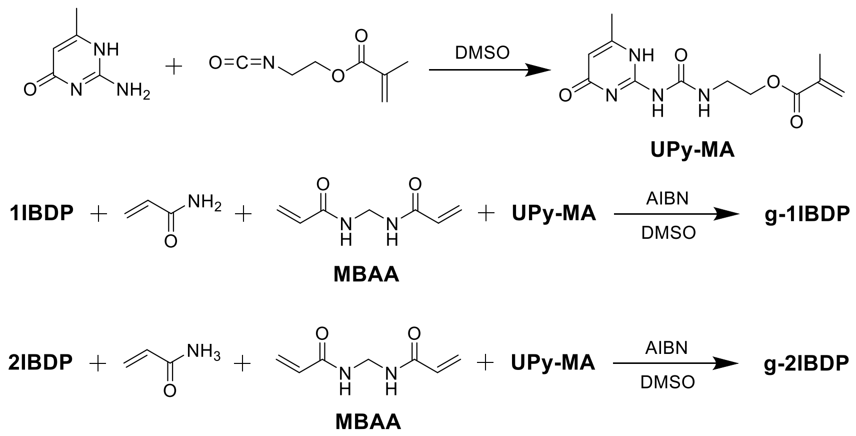

Scheme S3. Preparation routine of g-1IBDP and g-2IBDP.

Synthesis of UPy-MA. UPy-MA was synthesized according to literature procedure with same structure.[S2]

Synthesis of g-1IBDP and g-2IBDP. G-1IBDP and g-2IBDP were prepared by copolymerization of 1IBDP/2IBDP (1 eq), acrylamide (100 eq), MBAA (2 eq) and UPy-MA (2.5 eq) by radical polymerization with AIBN (0.1 eq) as the radical initiator at 70 ^{o}C under argon atmosphere in 1.3 mL DMSO. Gels were directly obtained after 24 h and washed with H_2O to remove the residual DMSO.

PHOTOPHYSICAL PROPERTIES

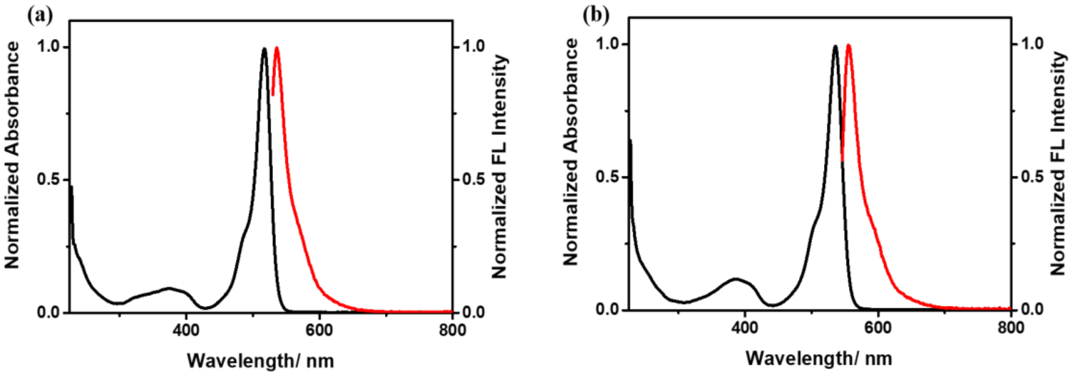

Figure S1. Normalized UV-Vis absorption spectra (black line) and fluorescence (red line) of (a) 1IBDP and (b) 2IBDP in dichloromethane. (c = 10^{-5} mol/L, λ_{ex1} = 518 nm, λ_{ex2} = 535 nm).

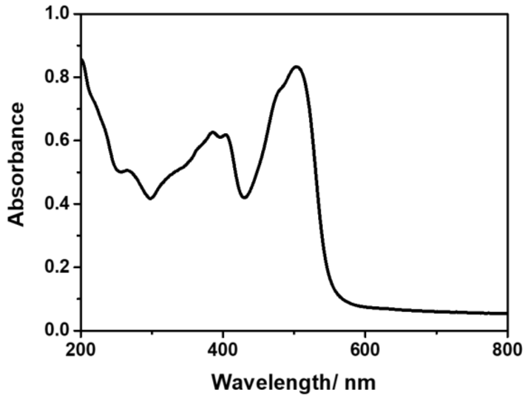

Figure S2. UV-Vis absorption spectra of p-BDP in amorphous solid state.

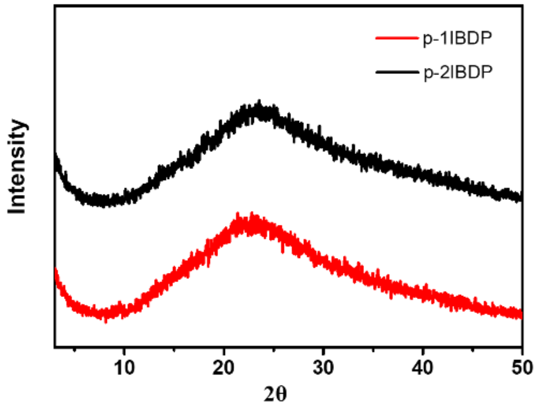

Figure S3. X-ray diffraction (XRD) patterns of p-1IBDP and p-2IBDP.

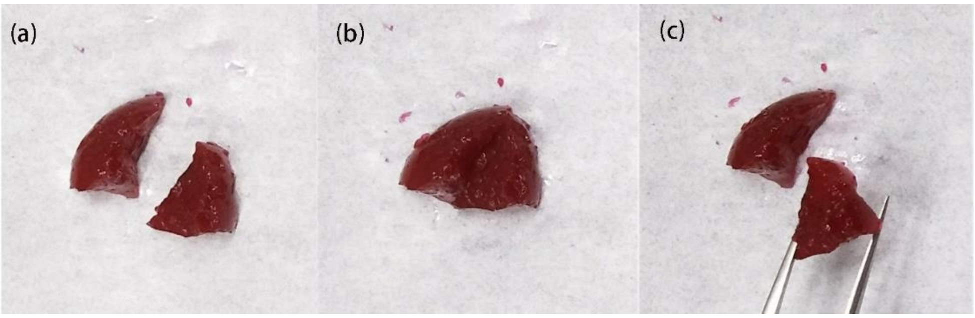

Figure S4. (a) Two pieces of reference gels composed of 2IBDP, acrylamide and MBAA (1:100:2). (b) The two pieces were put together. (c) The two pieces were easily separated by tweezers after being put together for 24 hours.

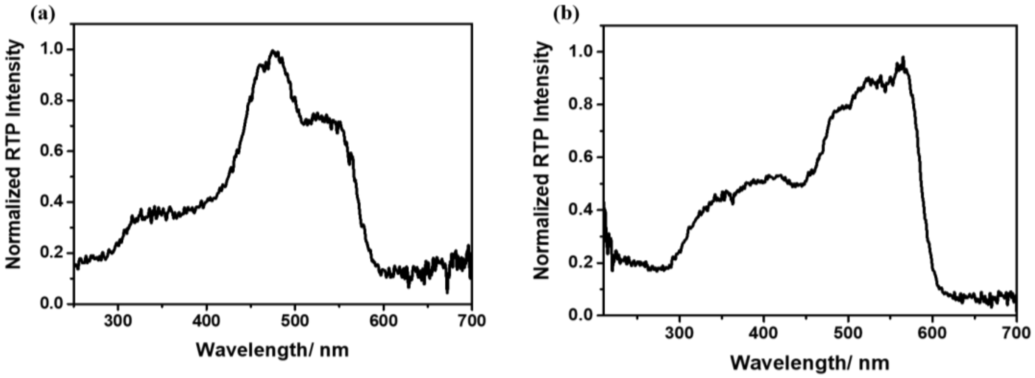

Figure S5. Excitation spectra of (a) g-1IBDP and (b) g-2IBDP (λ_{em} = 775 nm).

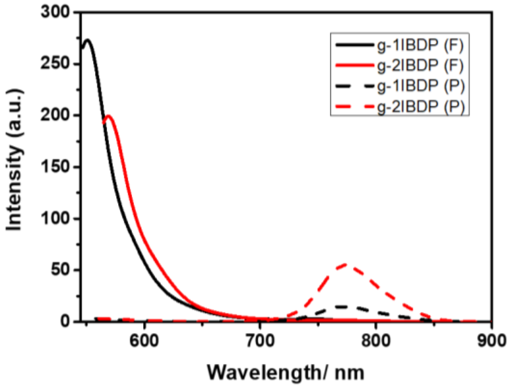

Figure S6. Fluorescence and RTP emission of g-1IBDP and g-2IBDP (λ_{ex1} = 520 nm, λ_{ex2} = 540 nm).

REFERENCES

(S1) N. G. Patil, N. B. Basutkar, A. V. Ambade, *Chem. Commun.*, 2015, **51**, 17708-17711.

(S2) X. Chang, Y. Geng, H. Cao, J. Zhou, Y. Tian, G. Shan, Y. Bao, Z. L. Wu, P. Pan, *Macromol. Rapid. Commun.*, 2018, **39**, 1700806.
